# Effects of Surgical Side and Site on Mood and Behavior Outcome in Children with Pharmacoresistant Epilepsy

**DOI:** 10.3389/fneur.2014.00018

**Published:** 2014-02-19

**Authors:** Elizabeth N. Andresen, Maya J. Ramirez, Kevin H. Kim, Ava B. Dorfman, Jennifer S. Haut, Patricia A. Klaas, Lara E. Jehi, Katherine Shea, William E. Bingaman, Robyn M. Busch

**Affiliations:** ^1^Department of Psychiatry and Psychology, Neurological Institute, Cleveland Clinic, Cleveland, OH, USA; ^2^Department of Psychology in Education, University of Pittsburgh, Pittsburgh, PA, USA; ^3^Cleveland Clinic Epilepsy Center, Neurological Institute, Cleveland Clinic, Cleveland, OH, USA

**Keywords:** mood, depression, anxiety, behavior, epilepsy surgery, children, pediatrics, neuropsychology

## Abstract

Children with epilepsy have a high rate of mood and behavior problems; yet few studies consider the emotional and behavioral impact of surgery. No study to date has been sufficiently powered to investigate effects of both side (left/right) and site (temporal/frontal) of surgery. One hundred patients (aged 6–16) and their families completed measures of depression, anxiety, and behavioral function as part of neuropsychological evaluations before and after surgery for pharmacoresistant epilepsy. Among children who had left-sided surgeries (frontal = 16; temporal = 38), there were significant interactions between time (pre to post-operative neuropsychological assessment) and resection site (frontal/temporal) on anhedonia, social anxiety, and withdrawn/depressed scales. Patients with frontal lobe epilepsy (FLE) endorsed greater pre-surgical anhedonia and social anxiety than patients with temporal lobe epilepsy (TLE) with scores normalizing following surgery. While scores on the withdrawn/depressed scale were similar between groups before surgery, the FLE group showed greater symptom improvement after surgery. In children who underwent right-sided surgeries (FLE = 20; TLE = 26), main effects of time (patients in both groups improved) and resection site (caregivers of FLE patients endorsed greater symptoms than those with TLE) were observed primarily on behavior scales. Individual data revealed that a greater proportion of children with left FLE demonstrated clinically significant improvements in anhedonia, social anxiety, and aggressive behavior than children with TLE. This is the first study to demonstrate differential effects of both side and site of surgery in children with epilepsy at group and individual levels. Results suggest that children with FLE have greater emotional and behavioral dysfunction before surgery, but show marked improvement after surgery. Overall, most children had good emotional and behavioral outcomes, with most scores remaining stable or improving.

## Introduction

Children with epilepsy are at higher risk of developing behavioral and emotional disturbances than children in the general population or those with other chronic illnesses (i.e., asthma, diabetes mellitus) (1–3). As such, the shared experience of chronic illness alone cannot account for the increased rate of behavioral and emotional difficulties in children with epilepsy. In fact, prevalence rates of psychopathology substantially increase from 7% in the general pediatric population to 29% in children with seizures and to 58% in children who have seizures with known structural brain abnormalities (1). Depression and anxiety are two of the most common psychiatric manifestations that often go untreated in children with intractable epilepsy (2, 4–6). In addition to emotional disruption, children with epilepsy also experience attention disorders and behavioral disturbances (e.g., hyperactivity, social withdrawal, aggression, and conduct problems) (4, 7). In fact, children with epilepsy experience behavioral disturbances about five times more often than children in the general population and three times more often than in those with other chronic medical conditions (8). In particular, children with temporal lobe epilepsy (TLE) have been found to experience more frequent emotional disturbances than children with extratemporal epilepsy (9). Therefore, behavioral and emotional morbidity in pediatric epilepsy is a substantial treatment concern.

Surgical intervention for intractable epilepsy during childhood has become a standard and increasingly effective treatment option. Rates of seizure-freedom following surgery for intractable pediatric epilepsy have been estimated between 59 and 80% (10). Historically, the primary aim of surgical intervention has been seizure remission or reduced seizure frequency. Surgical outcome has conceptually evolved and expanded over time to consider developmental, social, cognitive, psychological, and behavioral factors. Despite the acknowledged importance of these other factors in surgical outcome, most outcome studies continue to emphasize seizure-related factors (11–13). Few studies have evaluated behavioral and emotional outcome following pediatric epilepsy surgery. Given the prevalence of behavioral and emotional dysfunction and the standard use of surgical techniques to treat children with intractable seizures, a comprehensive investigation of behavioral and emotional outcome is essential.

To date, we are aware of only four published studies that have used standardized measures to assess emotional and behavioral change following epilepsy surgery in children (7, 9, 14, 15). These studies found improvements on various standardized parent-report mood and behavior inventories following surgery. Although different improvements were noted across the studies, reductions were generally seen on scales assessing internalizing symptoms, externalizing symptoms, attention problems, social interaction, thought problems, and hyperactivity. Notably, the small sample sizes in these studies did not allow for investigation of the effects of surgery side (right or left) or resection site (e.g., temporal or frontal), potentially masking important effects.

The goal of the present study was to extend the existing literature by evaluating behavioral and emotional outcome in a substantially larger sample of children following surgery for intractable childhood epilepsy. Specifically, our aim was to examine differences in outcome as a function of side and site of surgery at the group level as well as in individual patients.

## Materials and Methods

### Patients

This study involved an Institutional Review Board-approved, retrospective review of previously collected and archived data from children with medically intractable epilepsy, who were evaluated through the Neuropsychology Section at Cleveland Clinic as part of routine pre- and post-operative surgical investigations. Patients were included in the study, if they: (1) were between the ages of 6 and 16; (2) underwent a temporal lobe or frontal lobe resection for the treatment of intractable epilepsy between 1992 and 2012; (3) completed pre-surgical and post-surgical neuropsychological evaluations that included the measures of interest in this study; and (4) had no history of previous neurosurgery. From the available sample of 408 patients, a total of 100 children and adolescents met all inclusion/exclusion criteria. Patients were sequentially excluded from this study for the following reasons: age less than 6 years old (69 patients), did not undergo surgery (134 patients), resection site not frontal or temporal (5 parietal, 6 occipital, 45 multilobar), and did not complete post-surgical neuropsychological assessment (49 patients).

Patients included 51 males and 49 females with a mean age of 10.96 (SD = 2.84) and mean WISC-III or WISC-IV FSIQ of 84.12 (SD = 18.76). Mean duration of epilepsy was 5.27 years (SD = 3.61), and mean age at seizure onset was 5.81 (SD = 3.93). Patients were taking an average of 2.10 (SD = 0.83) antiepileptic medications at the time of their pre-operative neuropsychological assessment and an average of 1.68 (SD = 0.89) antiepileptic medications at the time of their post-operative assessment. Ninety-one percent of patients were Caucasian, and 89% were right-handed. Temporal lobe resections were conducted on 64 patients (38 left and 26 right) and frontal lobe resections were conducted on 36 patients (16 left and 20 right). A summary of demographic and seizure variables for study patients is provided in Table 1, separately by side and site of surgery.

**Table 1:** **Summary of demographic and seizure variables for study groups**.

	Left-sided		*F*/χ^2^	*p*	Right-sided		*F*/χ^2^	*p*
	Temporal *N* = 38	Frontal *N* = 16			Temporal *N* = 26	Frontal *N* = 20	
						
	Number (%)				Number (%)			
Gender			0.01	0.57			2.97	0.09
Male	16 (42)	7 (44)			13 (50)	15 (75)		
Female	22 (58)	9 (56)			13 (50)	5 (25)		
Race			4.54	0.34			2.85	0.24
Caucasian	33 (87)	15 (94)			24 (92)	19 (95)		
African American	3 (8)	0 (0)			0 (0)	1 (5)		
Hispanic/Latino	1 (3)	0 (0)			0 (0)	0 (0)		
Asian	0 (0)	1 (6)			2 (8)	0 (0)		
Other	1 (3)	0 (0)			0 (0)	0 (0)		
Handedness			0.74	0.84			0.13	1.00
Right	34 (89)	13 (81)			24 (92)	19 (95)		
Left	3 (8)	2 (13)			2 (8)	1 (5)		
Ambidextrous	1 (3)	1 (6)			0 (0)	0 (0)		
Seizure outcome at neuropsych follow-up			8.70	0.03			2.72	0.62
Engel I	35 (92)	10 (59)			23 (88)	16 (80)		
Engel II	2 (5)	2 (17)			1 (4)	0 (0)		
Engel III	1 (3)	2 (12)			0 (0)	1 (5)		
Engel IV	0 (0)	2 (12)			2 (8)	3 (15)		
Seizure outcome at last medical follow-up^c^			3.93	0.19			0.81	0.76
Engel I	20 (57)	7 (44)			17 (68)	11 (55)		
Engel II	0 (0)	0 (0)			0 (0)	0 (0)		
Engel III	14 (40)	6 (37)			7 (28)	8 (40)		
Engel IV	1 (3)	3 (19)			1 (4)	1 (5)		
Primary pathologies^b^								
Cortical dysplasia	11 (29)	12 (75)			7 (27)	12 (60)		
MTS	18 (47)	0 (0)			11 (42)	0 (0)		
Neuronal heterotopia	4 (11)	3 (19)			0 (0)	1 (5)		
Infarct	6 (16)	0 (0)			1 (4)	5 (25)		
Contusion	4 (11)	2 (13)			0 (0)	2 (10)		
Tumor	0 (0)	1 (6)			8 (31)	4 (20)		
Tuberous sclerosis	0 (0)	0 (0)			2 (8)	0 (0)		

	***M* (SD)**				***M* (SD)**	

Age	11.3 (3.1)	9.1 (2.4)	4.95	0.01	11.3 (2.7)	11.2 (2.5)	0.04	0.84
Age at seizure onset	5.6 (3.9)	4.3 (3.5)	1.41	0.24	7.1 (4.0)	5.5 (3.9)	1.8	0.18
Duration of epilepsy	5.7 (3.5)	4.9 (3.8)	0.58	0.45	4.3 (3.4)	6.2 (3.9)	3.31	0.08
Number of pre-surgical AEDs	2.1 (0.8)	2.3 (1.0)	0.50	0.49	1.8 (0.7)	2.3 (0.8)	3.72	0.06
Number of post-surgical AEDs^a^	1.6 (0.8)	1.8 (1.1)	1.07	0.31	1.6 (0.9)	1.9 (0.9)	1.12	0.30

*MTS = mesial temporal sclerosis; AEDs = antiepileptic medications*.

*^a^Number of post-surgical AEDs was available for all but two patients*.

*^b^Numbers do not sum to the total for each group as a number of patients had multiple pathologies*.

*^c^Long-term Engel scores were available for all but four patients*.

### Procedure

As part of routine comprehensive pre-operative and post-operative neuropsychological evaluations, children in this study and/or their parents completed the following questionnaires to assess behavior, mood, and anxiety difficulties: Achenbach Child Behavior Checklist – First or Second Edition (CBCL) (16), Children’s Depression Inventory (CDI) (17), and Revised Children’s Manifest Anxiety Scale – First or Second Edition (RCMAS) (18, 19). All measures were administered according to standard clinical procedures and normed according to the respective test manuals. For ease of interpretation, subscale names from the most recent editions of the CBCL and RCMAS are used hereinafter. The CBCL is a parent-report questionnaire evaluating a child’s behavioral and emotional functioning, social problems, and competencies. The following CBCL clinical subscales were used in the current study: anxious/depressed, withdrawn/depressed, somatic complaints, aggressive behavior, rule-breaking behavior, attention problems, thought problems, and social problems. The CDI is a self-report measure consisting of 27 statements, on each of which the child is instructed to select the response that best describes his/her feelings in the past 2 weeks. The following clinical subscales were investigated: negative mood, interpersonal problems, ineffectiveness, anhedonia, and negative self-esteem. The RCMAS is a self-report questionnaire used to measure anxiety in children ages 6–19. The following clinical scales were investigated: physiological anxiety, worry/oversensitivity, and social concerns. The CDI and RCMAS were administered as appropriate, taking into consideration each child’s reading ability and overall level of functioning. Children’s pre- and post-operative scores on the behavior, mood, and anxiety measures of interest in this study are presented in Tables 2 and 3.

**Table 2:** **Mean scores on mood, anxiety, and behavior measures for patients who underwent left-sided surgeries**.

	Temporal		Frontal	
	Pre-surgery	Post-surgery	Pre-surgery	Post-surgery
	*M* (SD)	*M* (SD)	*M* (SD)	*M* (SD)
**CBCL CLINICAL SCALES**				
Anxious/depressed	58.03 (7.83)	57.97 (8.32)	52.50 (3.48)	52.50 (3.67)
Withdrawn/depressed	57.59 (7.70)	58.68 (9.48)	59.43 (8.72)	54.86 (6.13)
Somatic complaints	58.88 (7.84)	55.56 (6.87)	56.57 (4.89)	55.86 (5.80)
Social problems	58.79 (8.37)	57.32 (8.86)	60.57 (4.89)	59.64 (10.98)
Thought problems	58.03 (8.89)	56.91 (7.98)	59.64 (7.61)	56.43 (7.67)
Attention problems	60.00 (9.52)	58.15 (9.75)	63.50 (9.26)	65.50 (13.01)
Rule-breaking behavior	54.29 (5.35)	53.82 (5.62)	55.07 (5.97)	55.07 (6.13)
Aggressive behavior	55.15 (6.37)	55.38 (8.55)	56.64 (6.97)	52.93 (4.34)
**CDI CLINICAL SCALES**				
Total depression	48.28 (7.98)	46.40 (7.90)	55.75 (10.47)	47.88 (9.96)
Negative mood	48.00 (9.86)	44.68 (6.01)	51.25 (10.40)	41.87 (5.41)
Interpersonal problems	49.00 (8.44)	48.60 (8.99)	52.75 (11.81)	52.75 (12.07)
Ineffectiveness	48.56 (10.15)	46.88 (8.18)	56.25 (10.44)	52.50 (14.23)
Anhedonia	50.36 (8.71)	50.25 (8.50)	59.25 (9.41)	50.25 (8.50)
Negative self-esteem	46.88 (7.98)	45.72 (6.20)	50.25 (6.16)	45.88 (10.15)
**RCMAS CLINICAL SCALES**				
Total anxiety	50.00 (14.21)	46.15 (10.21)	54.43 (7.55)	42.43 (10.63)
Physiological anxiety	47.27 (11.90)	44.42 (10.36)	49.57 (6.73)	45.71 (11.66)
Worry/oversensitivity	48.69 (13.07)	45.27 (9.46)	52.71 (9.96)	41.71 (8.42)
Social concerns	49.08 (12.15)	47.85 (7.72)	59.43 (7.66)	41.71 (8.04)

*CBCL = Children’s Behavior Checklist; CDI = Children’s Depression Inventory; RCMAS = Revised Children’s Manifest Anxiety Scale*.

**Table 3:** **Mean scores on mood, anxiety, and behavior measures for patients who underwent right-sided surgeries**.

	Temporal		Frontal	
	Pre-surgery	Post-surgery	Pre-surgery	Post-surgery
	*M* (SD)	*M* (SD)	*M* (SD)	*M* (SD)
**CBCL CLINICAL SCALES**				
Anxious/depressed	54.42 (7.43)	52.81 (4.64)	58.67 (9.08)	57.28 (8.48)
Withdrawn/depressed	56.19 (8.29)	53.81 (5.85)	62.78 (8.78)	60.83 (10.65)
Somatic complaints	55.54 (8.48)	55.69 (7.31)	61.28 (9.00)	57.11 (7.28)
Social problems	58.58 (9.74)	56.08 (7.58)	69.44 (12.40)	63.72 (9.81)
Thought problems	58.42 (10.72)	54.69 (6.23)	63.72 (7.28)	59.22 (8.00)
Attention problems	59.58 (11.19)	57.65 (9.83)	69.00 (10.53)	64.39 (9.97)
Rule-breaking behavior	52.81 (4.39)	52.88 (4.34)	57.22 (7.79)	56.78 (8.54)
Aggressive behavior	54.81 (6.17)	53.81 (6.88)	60.28 (9.35)	58.50 (7.59)
**CDI CLINICAL SCALES**				
Total depression	48.00 (9.78)	46.18 (9.11)	54.54 (12.72)	51.08 (8.32)
Negative mood	50.68 (12.69)	47.27 (6.92)	53.54 (12.79)	52.69 (11.84)
Interpersonal problems	49.55 (12.46)	47.64 (12.22)	49.00 (6.25)	49.08 (10.56)
Ineffectiveness	47.45 (9.51)	46.73 (8.81)	53.23 (12.96)	50.92 (10.79)
Anhedonia	51.36 (12.63)	48.14 (10.34)	55.54 (9.72)	52.23 (8.11)
Negative self-esteem	42.59 (4.82)	45.50 (7.40)	52.23 (14.23)	48.77 (7.72)
**RCMAS CLINICAL SCALES**				
Total anxiety	48.64 (11.22)	46.77 (14.99)	56.20 (14.38)	50.73 (11.02)
Physiological anxiety	47.64 (10.33)	46.77 (12.54)	54.87 (14.08)	48.73 (13.39)
Worry/oversensitivity	47.14 (11.60)	48.00 (13.28)	53.60 (10.63)	46.80 (11.14)
Social concerns	48.24 (12.66)	47.33 (11.37)	55.29 (12.26)	51.43 (11.61)

*CBCL = Children’s Behavior Checklist; CDI = Children’s Depression Inventory; RCMAS = Revised Children’s Manifest Anxiety Scale*.

Post-surgical neuropsychological evaluations were conducted on average 8.5 months (SD = 12.7) following surgical resection for treatment of intractable seizures and 10.6 months (SD = 6.2) following the pre-operative neuropsychological evaluation. At the time of post-surgical neuropsychological assessment, seizure outcomes were as follows: Engel class I (84 patients), Engel class II (5 patients), Engel class III (4 patients), and Engel class IV (7 patients). Longer term seizure outcome data (mean = 62 months after pre-operative neuropsychological evaluation; range = 12–223 months) were available on a large subset of patients (*n* = 96). In patients who underwent temporal resections, 37 had Engel class I, 21 had Engel class III, and 2 had Engel class IV. In patients who underwent frontal resections, 18 had Engel class I, 14 had Engel class III, and 4 had Engel class IV.

### Data analyses

First, patients were categorized into groups based on resection site (frontal, temporal). Then, one-way ANOVAs and chi-square tests were conducted to examine potential group differences on relevant demographic and seizure variables separately by side of surgery (left, right). Next, a series of repeated measures ANCOVAs (left-sided surgeries controlled for age) or ANOVAs (right-sided surgeries) were performed using bootstrapping to adjust for influential cases and small sample size. One thousand replications were performed, and bootstrap-adjusted *p*-values were examined. ANCOVAs or ANOVAs were performed on each of the behavioral and emotional scales described above (i.e., eight CBCL clinical subscales, five CDI clinical subscales, and three RCMAS anxiety subscales) as a function of site of resection (temporal, frontal) over time (pre-surgical, post-surgical). All analyses were conducted separately for patients who underwent left versus right-sided surgeries. Summaries of mean subscale scores for each of the patient groups, separately by surgical side, are provided in Tables 2 and 3. Given that patients who are left-handed have a higher likelihood of having atypical language lateralization, all analyses were re-run after excluding patients who were right-hemisphere dominant (*n* = 1) or who had not had any lateralizing procedure completed (*n* = 3). The overall pattern of results remained the same.

Finally, to examine post-operative changes in mood and behavior at the individual patient level, change scores were calculated for each patient on each scale by subtracting their post-surgical scores from their pre-surgical scores. A series of chi-square analyses with exact test were then conducted to examine potential differences in clinically meaningful change (decline, no change, improvement) as a function of site of surgery (frontal, temporal) separately in patients who underwent left versus right-sided resections. Change scores were considered to be clinically meaningful if they were greater than or equal to one standard deviation (i.e., ±10 points).

## Results

### Left-sided resections

#### Demographics

One-way ANOVAs and chi-square tests (Table 1) revealed significant differences in age [*F*(1, 53) = 4.95, *p* = 0.01, $\eta_{p}^{2}=0.11$] and seizure outcome [χ^2^(3) = 8.70, *p* = 0.03, Φ = 0.42] between children who underwent temporal versus frontal lobe resections. Specifically, the TLE group was approximately 2 years older and had a higher rate of seizure-freedom following surgery than the FLE group. Subsequent analyses included age as a covariate. However, given the strong relationship between seizure outcome and resection site, with better outcomes reported following temporal compared to frontal lobe resections as well as the very small sample of patients who were not seizure-free at post-operative follow-up (20, 21), this variable could not be adequately controlled statistically.

#### CDI clinical scales

Repeated measures ANCOVAs revealed a group × time interaction on the anhedonia subscale, *F*(1,30) = 7.82, bootstrap-adjusted *p* = 0.01, $\eta_{p}^{2}=0.22.$ Specifically, children in the FLE group reported more symptoms pre-surgically, with post-operative symptom improvement. No main effects were found. The descriptive statistics are reported in Table 2.

#### RCMAS clinical scales

An interaction was seen on the social concerns subscale, *F*(1,31) = 17.14, bootstrap-adjusted *p* < 0.00, $\eta_{p}^{2}=0.35.$ Again, the FLE group had higher scores pre-surgically which normalized post-operatively. No main effects were found.

#### CBCL clinical scales

**Figure 1:**
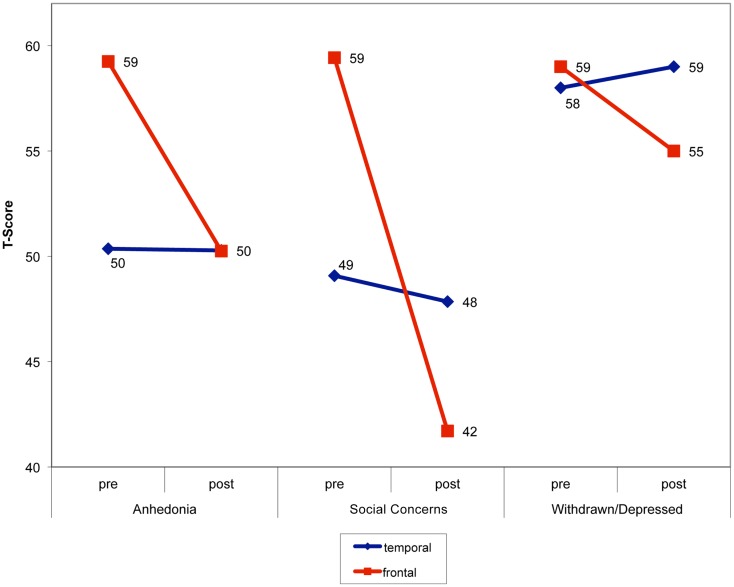
**Change in mood, anxiety, and behavior in children following left-sided surgery, with children who underwent frontal resections showing significant improvement**.

A group × time interaction was also observed on the withdrawn/depressed subscale, *F*(1,46) = 4.07, bootstrap-adjusted *p* = 0.04, $\eta_{p}^{2}=0.08.$ Results indicated that the FLE group’s scores improved following surgery, while the TLE group’s scores remained largely stable. A main effect of group was observed on the anxious/depressed subscale, such that the TLE group had higher scores on this index than the FLE group collapsed across time, *F*(1,45) = 6.48, *p* = 0.01, $\eta_{p}^{2}=0.15.$ All significant interactions are depicted in Figure 1.

### Right-sided resections

#### Demographics

There were no significant group differences on demographic or seizure variables among patients who underwent right-sided surgery (Table 1). Therefore, no covariates were used in subsequent analyses.

#### CDI clinical scales

No significant interactions were seen; however, there was a main effect of group on the negative self-esteem subscale such that children in the FLE group had higher scores than the TLE group on this subscale collapsed across time, *F*(1,33) = 6.79, *p* = 0.04, $\eta_{p}^{2}=0.17.$ The descriptive statistics are reported in Table 3.

#### RCMAS clinical scales

No significant interactions or main effects were seen on any of the subscales from the RCMAS in children who underwent right-sided resections.

#### CBCL clinical scales

No significant interactions were found on the CBCL. Main effects of time were observed on the following scales: withdrawn/ depressed, *F*(1,42) = 5.23, bootstrap-adjusted *p* = 0.03, $\eta_{p}^{2}=0.13,$ social problems, *F*(1,42) = 14.83, bootstrap-adjusted *p* = 0.00, $\eta_{p}^{2}=0.27,$ thought problems, *F*(1,42) = 11.04, bootstrap-adjusted *p* = 0.00, $\eta_{p}^{2}=0.25,$ and attention problems, *F*(1,42) = 10.62, bootstrap-adjusted *p* = 0.02, $\eta_{p}^{2}=0.21.$ Specifically, when collapsed across surgery groups, children endorsed fewer symptoms on these measures post-operatively. Main effects of group were also observed on a number of CBCL scales including anxious/depressed, *F*(1,42) = 4.69, *p* = 0.05, $\eta_{p}^{2}=0.09,$ withdrawn/depressed, *F*(1,42) = 8.26, *p* = 0.01, $\eta_{p}^{2}=0.15,$ social problems, *F*(1,42) = 10.87, *p* = 0.01, $\eta_{p}^{2}=0.21$, thought problems, *F*(1,42) = 4.84, *p* = 0.03, $\eta_{p}^{2}=0.10,$ attention problems, *F*(1,42) = 7.09, *p* = 0.01, $\eta_{p}^{2}=0.15,$ and aggressive behavior, *F*(1,42) = 6.06, *p* = 0.03, $\eta_{p}^{2}=0.12.$ Specifically, patients with FLE demonstrated higher scores on these subscales, collapsed across time, than patients with TLE.

### Individual change scores

**Figure 2:**
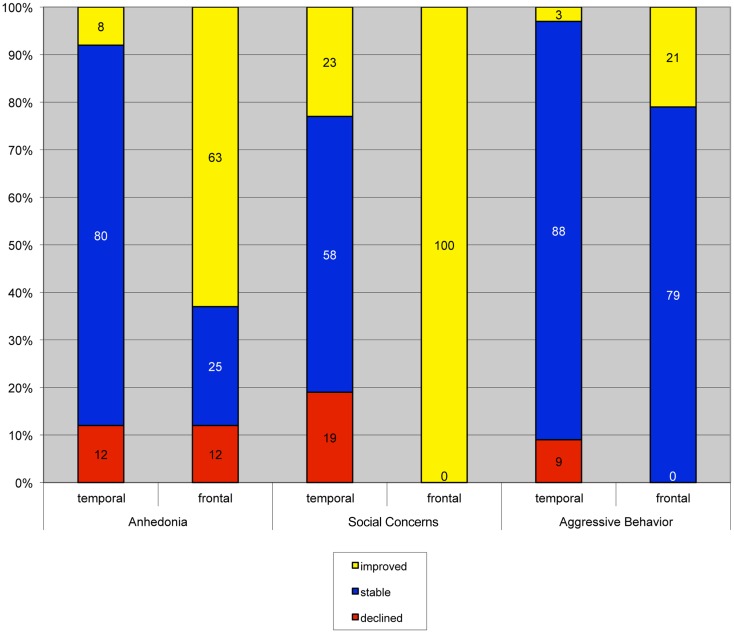
**Clinically meaningful change on an individual level in children who underwent left-sided surgeries**. Children who received frontal resections had significantly higher rates of clinically significant improvement than children who underwent temporal resections.

In children who underwent left-sided surgery, there was a significant difference in the proportion of patients demonstrating clinically meaningful post-operative change as a function of surgical site on the CDI anhedonia scale, χ^2^(2) = 11.24, *p* = 0.00, Cramer’s *V* = 0.58, and the RCMAS social concerns scale, χ^2^(2) = 13.67, *p* = 0.00, Cramer’s *V* = 0.64. There was a similar trend on the aggressive behavior subscale of the CBCL, χ^2^(2) = 5.41, *p* = 0.06, Cramer’s *V* = 0.34. Specifically, post-operative improvements on these scales were observed more frequently among children with FLE than those with TLE (Figure 2).

No significant differences were found in children who underwent right-sided surgery, although a trend was noted on the CDI negative self-esteem subscale, χ^2^(2) = 5.75, *p* = 0.05, Cramer’s *V* = 0.41. Again, post-operative improvements were noted somewhat more frequently in the FLE group.

## Discussion

The current investigation demonstrates differences in post-surgical emotional and behavioral outcome as a function of surgical side and site in a large sample of pediatric patients with epilepsy. Children with left FLE had higher pre-surgical scores on the CDI scale of anhedonia and the RCMAS scale of social anxiety than children with left TLE. However, mean scores among FLE patients improved following surgery to the degree that post-operative symptom endorsements were similar to those of the TLE group. An additional interaction was noted on the CBCL withdrawn/depressed scale: While group scores were similar prior to surgery, the left FLE group demonstrated greater improvement in symptoms following surgery than the left TLE group. We are aware of only four prior studies (7, 9, 14, 15) that have examined mood and/or behavior outcome following epilepsy surgery in children based on objective self- or parent-report measures. Consistent with our findings, all of these studies found post-operative improvements in aspects of mood and/or behavior. The only study to examine potential localization effects (7) did not find significant differences in behavior outcome in children who underwent temporal versus extratemporal resections. This discordant finding may be largely related to differences in sample composition; specifically, more children with FLE were included in the present study. The majority of patients in Lendt’s extratemporal group had posterior resections, and only four patients in that group had resections within the frontal lobe.

Interestingly, the interaction effects observed between surgical site and time in the current study were only observed in children who underwent left-sided surgeries. This finding is consistent with existing literature. Numerous studies over the years have demonstrated differential effects of lesion laterality on mood in adult clinical populations such that mood difficulties are most frequently associated with anterior lesions in the dominant hemisphere (22). Lateralized findings have rather consistently been observed in adult patients with epilepsy as well, with left-sided seizure foci associated with greater depressive symptomatology than right-sided seizure foci (23–27). Few studies have examined laterality effects on mood, anxiety, and behavior in children (28–30), and only two known studies have attempted to examine the effects of surgical side on post-operative psychological status in pediatric patients. Williams et al. (14) did not find any significant differences in post-operative mood, anxiety, or behavior between children after left versus right temporal lobectomies. However, their sample included only nine patients and was therefore underpowered. McLellan et al. (15) did not find significant differences in mood or anxiety disorders between children who underwent left versus right temporal lobectomies, but identified higher post-operative rates of developmental disorders (e.g., pervasive development disorder) and attention-deficit/hyperactivity disorder after right compared to left temporal lobectomies.

While no significant interaction effects between site of surgery and time were observed in children who underwent right-sided resections, several potentially important main effects were observed. First, parents of children with right FLE reported more mood symptoms and greater behavioral dysfunction on a number of CBCL syndrome scales than parents of children with TLE. Thus, these findings support the notion that anterior lesions have more profound effects on mood/behavior (22). Moreover, behavioral disturbances secondary to impulsivity and disinhibition are often associated with damage to the frontal lobes (31, 32). Second, main effects of time were observed on many of the same CBCL scales (i.e., withdrawn/depressed, social problems, thought problems, and attention problems) with post-operative improvement in symptoms across groups following right-sided resections. These findings are quite consistent with prior pediatric studies (7, 9, 14) that demonstrated improvements in behavior after epilepsy surgery. It is interesting that improvements in behavior and mood were limited to the parent-report measure. Similar improvements in functioning were not observed on self-report measures of mood and anxiety completed by the children themselves. This apparent difference in perspective between children and their parents may potentially reflect a lack of awareness of post-surgical emotional and behavioral change among children who undergo right-sided resections (33, 34). More research will be required to investigate this possibility.

Studies in the adult epilepsy literature have demonstrated that mood improvement is related to surgical outcome (35, 36). Unfortunately, given the strong relationship between surgical site and seizure outcome and the small sample size of individuals with poor seizure outcome at the time of post-operative follow-up, we were unable to adequately control for the potential effects of seizure outcome on mood change following surgery. Nevertheless, the left FLE group in our sample demonstrated greater post-operative mood improvements than patients with TLE, despite poorer seizure outcomes. This suggests that seizure-free outcome was not the driving force behind the post-operative improvements observed in children with FLE. It is interesting that the expected differences in seizure outcome (i.e., better outcomes following temporal versus frontal resections) were only observed following left-sided surgeries. We have observed similar findings in our adult surgical samples and hypothesize that this may be related to specific location and extent of surgical resection (21). Surgeons are often able to be more aggressive in attempting to remove seizure foci within the non-dominant hemisphere whereas surgeries within dominant frontal and temporal lobe regions may be more limited to avoid impingement into eloquent cortex. Future studies will be required to examine this further.

One of the limitations of prior research on this topic is that most data analyses are limited to examining differences in group means with little attention paid to potential differences in mood or behavior change at the individual level. The current study found that when change is examined in individuals, patients with left FLE were more likely than their TLE counterparts to show clinically significant improvement on several mood and behavior scales following surgery. The differences in the proportion of individual improvements were most apparent on the RCMAS social concerns scale and CDI anhedonia scale, where interactions were seen in the group data. In fact, all of the patients with left FLE reported clinically significant post-surgical improvement on the social concerns scale (range 12–32 *T*-score point improvement post-operatively), and 63% of that group reported clinically significant improvement on the anhedonia scale (range 15–19 *T*-score points). This is quite striking and suggests that, in this sample, surgery for left FLE had a powerful effect on these aspects of mood and anxiety. Notable improvements were also observed on the CBCL aggressive behavior subscale among these patients. Further examination of individual data on this subscale revealed that none of the children with TLE who had clinically significant problems with aggression improved significantly following surgery, while several children in the FLE group demonstrated clinically significant post-surgical improvement on aggression scores. However, it is important to note that pre-surgically, none of the children who demonstrated clinically significant change on this subscale had scores in the clinically significant range (i.e., *T*-score above 60).

While group mean scores on the measures used in this study were generally in the normal range (Tables 2 and 3), examination of individual patient data revealed a number of patients with clinically elevated mood and behavior problems, highlighting the importance of including mood and behavior inventories as part of standard pre-operative investigations to identify these children and inform treatment recommendations. This also demonstrates the way in which group data can be misleading and obscure potentially important individual differences. Further, while changes of several points may not appear impressive from a quantitative perspective, from a qualitative perspective such seemingly small changes can have a large impact on quality of life (e.g., there may be a large qualitative difference between the 1-point CDI answer “Many bad things are my fault” and the 0-point answer “Bad things are not usually my fault,” or “I feel alone many times” versus “I do not feel alone”).

Although the left-sided FLE group showed the most benefit on both the group level and at the individual level, examination of the data shows that most children showed clinically significant improvement or had stable scores across measures regardless of surgical resection site, and rates of seizure-freedom did not differ in children whose scores improved, remained stable, or declined. However, the small percentage of children who demonstrated clinically significant post-operative declines on several scales must not be overlooked. Future studies will be required to better characterize the factors that place this small subset of children at risk for worsening mood and/or behavior issues following epilepsy surgery. Interestingly, review of post-operative neuropsychological reports for children who showed mood declines after surgery revealed that for about half of these patients this decline appeared to be in response to difficulty with a recent transition (i.e., transition to a more difficult stage in school, transition away from the sick role) rather than surgery *per se*.

Several limitations to the current study deserve mention. Given that there was not a non-surgical control group included in this study, we cannot definitively conclude that the interaction and time effects observed in this study are the result of the surgery itself and not simply the passage of time. However, if passage of time were the primary factor related to change, one would not expect differences in mood/behavior outcome as a function of side and site of resection. Nevertheless, future studies that include control patients will be necessary to confirm that our findings are indeed related to surgery. Information regarding the relationship between extent of resection and post-operative mood/behavior outcome was not examined. The location and extent of surgical resection likely plays an important role in outcome in this regard, particularly among patients who undergo frontal lobectomies. Future studies will be required to investigate the potential impact of resection extent and effects of different resection sites within the frontal lobe on various aspects of mood and anxiety. Additionally, it is known that antiepileptic medications can negatively affect mood and behavior in children (37–40). Unfortunately, limited data were available regarding the types and dosage of antiepileptic medications the patients in this study were taking at the time of their neuropsychological evaluations. Information regarding other medications that may impact mood and behavior (e.g., antidepressants, anxiolytics, stimulants) was also unavailable. Future studies should investigate the potential impact of medication type/dose on the magnitude and direction of post-surgical changes in emotional and behavioral functioning. The current study had a relatively brief follow-up period of approximately 8 months following surgery. Studies with longer follow-up will be needed to determine whether the observed improvements are maintained over a longer period of time, when Engel I rates are likely to be lower. Finally, this study is limited to some degree by the relatively small final sample size after the sample was divided by side and site of surgery. We attempted to control for this limitation by conducting bootstrapped ANOVAs, which allowed us to have greater confidence that our findings are meaningful. However, while the sample is relatively small by statistical standards, this represents the largest known pediatric surgical sample in which mood/behavior outcome has been examined. These data were obtained from a registry including all pediatric epilepsy patients who completed pre/post neuropsychological testing with the measures of interest in this study at Cleveland Clinic between 1992 and 2012. Studies using larger samples will likely require multicenter collaboration.

In sum, these results suggest generally favorable psychological outcomes in pediatric patients following epilepsy surgery. Neuropsychologists and other providers at epilepsy centers are often asked by parents of children being evaluated for epilepsy surgery whether there will be significant changes in their child’s mood, behavior or personality after surgery. To date, there has been little research to support our clinical sense that most children do well following surgery. The current study provides reassurance that mood and behavior outcomes in most children are quite favorable based on both group and individual data.

## Conflict of Interest Statement

The authors declare that the research was conducted in the absence of any commercial or financial relationships that could be construed as a potential conflict of interest.
